# Case study: lessons learned from a community-based early frailty intervention programme in Singapore

**DOI:** 10.3389/fpubh.2024.1445347

**Published:** 2024-10-23

**Authors:** Kamala Priya Jayaprakash, Rachel Ngo, Elianna Lee, Pey Ling Chng, Hengky Lee, Salan Chua, Deborah Lee, Carina Wong, Viknessh S

**Affiliations:** ^1^National Preventive Medicine Residency Programme, National University Health System, Singapore, Singapore; ^2^Health Promotion Board, Singapore, Singapore; ^3^Tan Tock Seng Hospital, Singapore, Singapore

**Keywords:** frailty, older adults, early frailty intervention, community-based programmes, strength and balance

## Abstract

Frailty is a dynamic and evolving state of health which involves the gradual loss of physiological in-built reserves. In Singapore, there is growing interest in delivering frailty intervention programmes at scale in the community to meet the demands of an ageing population. New methods of programme delivery such as community-led models that do not rely on healthcare professional manpower are critical to address this unmet need. In this paper, we describe our experience and some lessons learned from the implementation of a community-based early frailty intervention programme for older adults, delivered for the first time by trained laypersons. From August to September 2022, “Steady Lah,” a community-based early frailty intervention programme with physical activity and nutrition-based elements, was conducted at an Active Ageing Centre in Singapore. A total of 23 participants with mean age of 73.8 years were enrolled in the 12-session programme comprising of progressive strength and balance-based exercises and workshop-based learning focusing on nutrition. In the implementation of this run of “Steady Lah,” modifications were made from a healthcare professional-led model to a trained layperson-led model with additional steps taken to ensure participant safety and assess overall effectiveness when delivered by trained laypersons. Good collaboration between stakeholders in healthcare institutions and the community is necessary to co-develop a model that prioritises the needs of the frail older adults.

## 1 Introduction

Frailty is defined as a dynamic and evolving state of health which involves the gradual loss of physiological in-built reserves leading to losses in one or more domains of human function (physical, cognitive, psychological and/or social) and increases the vulnerability of older adults to adverse health-related outcomes (1).It is associated with adverse outcomes such as mortality, increased hospitalisation, disability, and falls (2).

In Singapore, frailty is an area of focus at the national level. With a rapidly ageing population and an estimated prevalence of pre-frailty and frailty at 30% and 5% respectively, there is growing interest in enhancing nationwide efforts to address the needs of this specific population of older adults (3, 4). In May 2023, the Singapore Ministry of Health published a “National Frailty Strategy Report” (1). One of the key recommendations in the report was for the community to play a bigger role in both identification of older adults with frailty, and management of older adults with early frailty through interventions such as exercise, nutritional support and falls prevention.

Current evidence shows that frailty can be prevented, reversed or delayed in the early stages and managed in later stages, through early detection and interventions to optimise functional ability, activity participation and quality of life (1). Examples of effective interventions include multicomponent exercises that incorporate elements of strength, resistance, balance, and aerobic capacity, and multi-domain interventions focusing on other areas such as nutrition (5–8).

Various frailty intervention programmes have been developed and implemented by healthcare institutions in Singapore. One example is a programme named “Steady Lah,” loosely named after a local Singlish expression that is typically used to praise someone. “Steady Lah” was first conceptualised in 2018 by healthcare professionals from Tan Tock Seng Hospital (TTSH), one of the largest multi-disciplinary hospitals in Singapore. The programme aimed to improve participants’ overall physical function, delay the progression of frailty, and was delivered by healthcare professionals such as physiotherapists and dieticians. Initial runs of “Steady Lah” conducted at selected community sites in 2019 showed positive results. However, the programme design and healthcare professional-dependent model of delivery meant that there were challenges with scaling up the programme to more sites to meet the overall demand in the community. In November 2021, the Health Promotion Board (HPB), a statutory board under the Ministry of Health with the vision of building “A Nation of Healthy People,” partnered TTSH to jointly review the curriculum and programme design of “Steady Lah” with intent to scale up nationwide. A revised model of this community-based early frailty intervention programme delivered for the first time by trained laypersons, was implemented in 2022. This case study documents the experience of our team and shares lessons learned for future applications.

## 2 Context

### 2.1 Setting

The revised model of “Steady Lah” was implemented at an Active Ageing Centre (AAC) in the central region of Singapore from August to September 2022. AACs are drop-in social recreational centres managed by not-for-profit organisations, which extend support to older adults living nearby in the community (9). There are currently 157 AACs located across the island in Singapore, and this is planned to increase to 220 by 2025 (10).This would ensure that 8 in 10 older adults would have an AAC near their homes and be able to benefit from the activities offered (10). Examples of activities in an AAC include physical activity programmes, social events like singing and games, communal meals, and organised excursions to cater to the wide range of interests of older adults. Traditionally the activities are catered to older adults with varying functional abilities rather than specifically designed for sub-groups such as older adults with early frailty. For our programme, interested participants at the AAC were referred to attend the programme registration day by AAC managers based on a description of the programme objectives and target population of older adults with early frailty. The final assessment on eligibility was conducted by the team from the HPB’s Healthy Ageing Division prior to enrolment.

### 2.2 Population

The target population for the programme was community-dwelling older adults aged 50 and above who were at-risk or showing early signs of frailty. We used gait speed as a proxy measure for early frailty and participants with gait speeds between 0.9 m/s to 1.2 m/s were assessed to be eligible for the programme. This cut-off range was decided in reference to proposed cut-offs and gait speed norms available in regional and local literature, and identified older adults with average or lower gait speeds.

For reference, the lowest quintile of gait speed in the local population was reported to be 0.9 m/s (11). This is slightly lower but close to the recommendation of the 2019 Asian Working Group for Sarcopenia where a gait speed cut-off of 1.0 m/s is used to identify low physical performance (12). The upper range of eligibility of the programme was set at 1.2 m/s. This was referenced from the same local study which found that the average gait speed for older adults aged 51 to 80 ranged from 0.9 m to 1.14 m/s (11). In a similar study conducted among older Japanese adults aged 65 and above, the 3rd quintile for usual gait speed in men was 1.25–1.34 m/s while that in women was 1.20–1.31 m/s (13). The upper limit of 1.20 m/s was set to exclude more robust older adults who may not benefit as much from the “Steady Lah” programme.

In addition, participants were excluded if they required assistance to ambulate at home, were undergoing chemotherapy or radiotherapy, were undergoing dialysis, or were diagnosed with kidney disease or Parkinson’s disease. This was because certain physical exercises in the programme curriculum were unsuitable for participants with these conditions or because the general nutritional advice provided during workshops was not appropriate for participants on restricted diets or specific medications.

A total of 34 older adults were assessed on registration day. Of these, 23 were identified to be eligible for the programme. An overview of the age range and gender distribution of the participants is described in Table 1.

**Table 1:** Baseline characteristics of participants

Variable	Participants assessed and recruited		Participants assessed to be unsuitable		All participants assessed	
	Mean +/− SD or %	*N*	Mean +/− SD or %	*N*	Mean +/− SD or %	*N*
Age	73.8 +/− 7.52	23	73.5 +/− 7.20	11	73.7 +/−7.41	34
Gender						
Male	8.7%	2	9.1%	1	8.8%	3
Female	91.3%	21	90.9%	10	91.2%	31

## 3 Programme description

“Steady Lah” is a community-based early frailty intervention programme with physical activity and nutrition-based elements conducted across 12 sessions. It was conducted two times a week on Mondays and Thursdays for 2 h. Each session comprises of 60 min of physical activity and 60 min of workshop-based learning. The revised model was delivered by trained laypersons including fitness trainers and workshop facilitators from August to September 2022. The 60-min physical activity element was of low to moderate intensity and focused on progressive strength and balance exercises. Some examples of exercises in the programme curriculum include sit to stand, heel raises, and side steps. The difficulty of the exercises was adjusted according to the performance of the participants by varying the number of repetitions, the range of motion or the level of hand support used during the exercises. Taking the example of heel raises, the participants are first introduced to six repetitions of bilateral heel raises with hand support on a chair placed in front. When the instructor assesses that most of the class can perform the required number of repetitions of this exercise, the number of repetitions is then increased to eight or 10. The subsequent increase in difficulty for this exercise type is when participants perform bilateral heel raises without hand support and then, unilateral heel raises with hand support.

**Figure 1:**
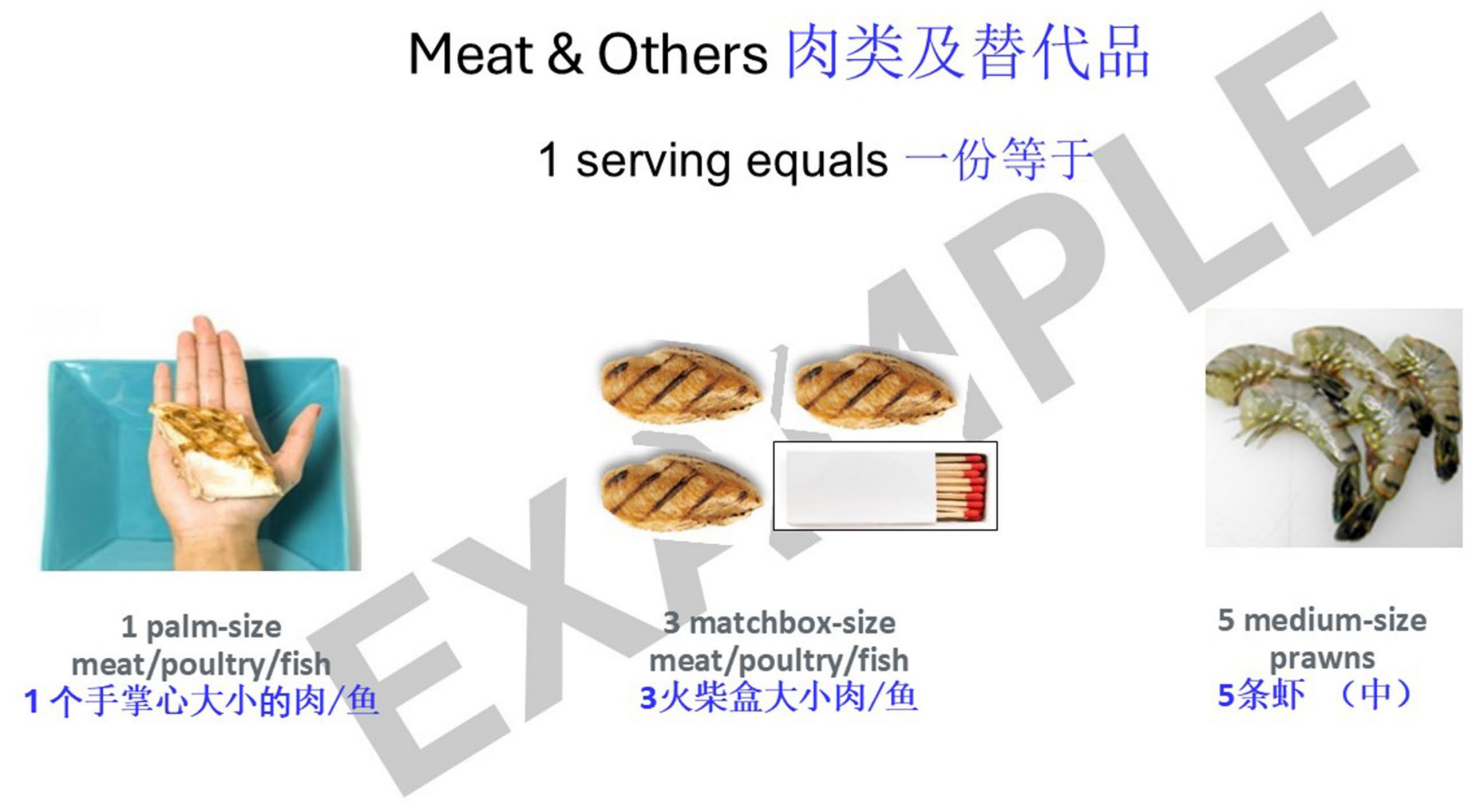
Example of “Steady Lah” workshop content—protein serving size.

**Figure 2:**
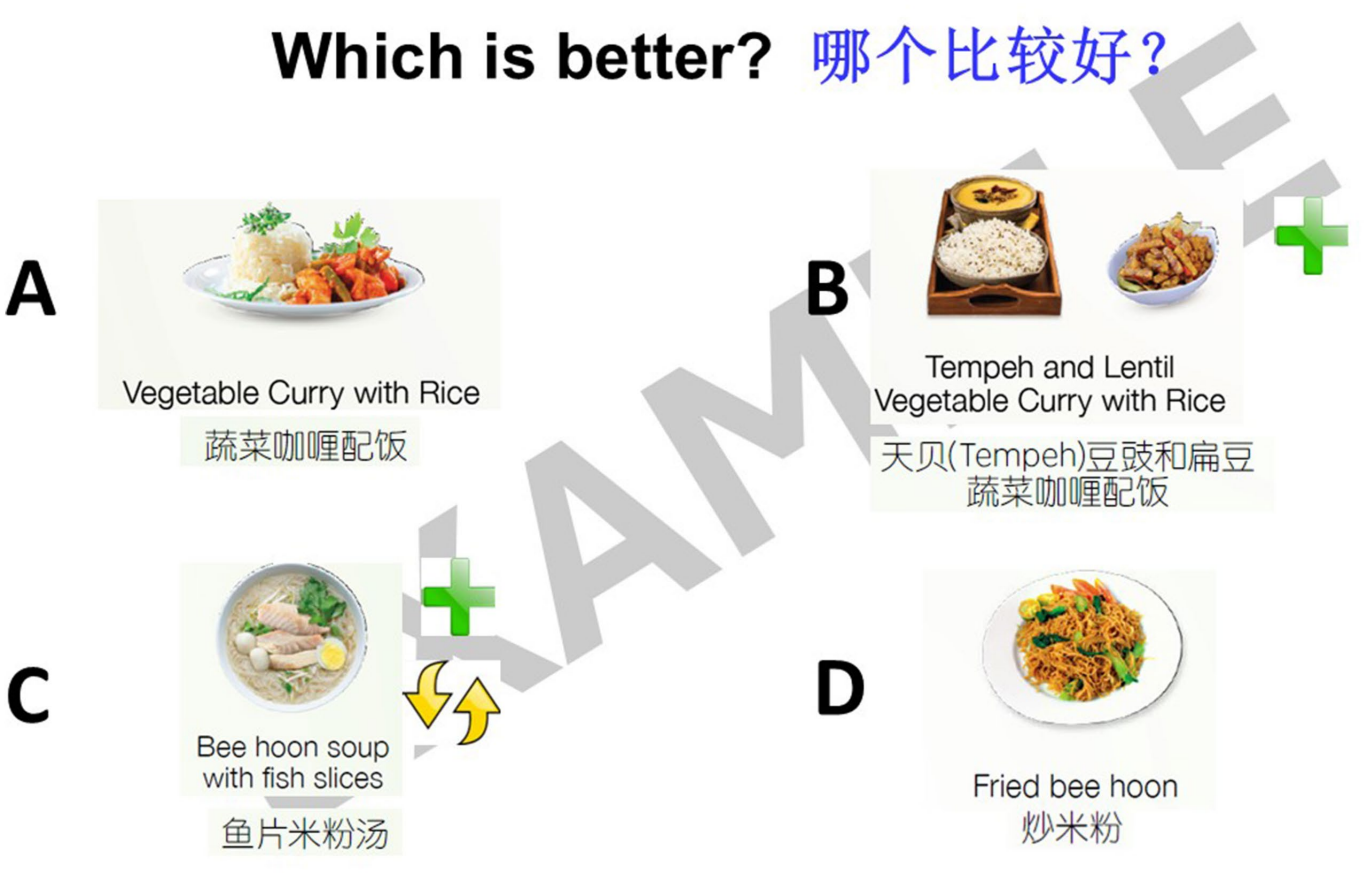
Example of “Steady Lah” workshop content—incorporating protein in meals.

The workshop component included short presentations and interactive activities to educate older adults on tips for a healthy diet with emphasis on adequate protein and calcium intake. This was conducted by a bilingual trainer in both English and Mandarin. For the message of ensuring adequate protein intake, the presentation component included material on the definition of protein, the importance of protein to maintain physical function and reduce muscle loss, the recommendation to consume protein at each meal, and common protein-rich foods. In the interactive activities, participants would identify which food item was rich in protein among the images flashed or identify which serving size of a particular protein-rich food item was equivalent to one serving of protein. Some examples of the material shared during the workshops on protein serving size and modifying meals to increase protein content are as illustrated in Figures 1, 2. A modified food frequency questionnaire developed by TTSH to capture food groups relevant in the local setting among older adults was also introduced as a tool for older adults to understand their protein and calcium intake and make incremental changes.

**Figure 3:**
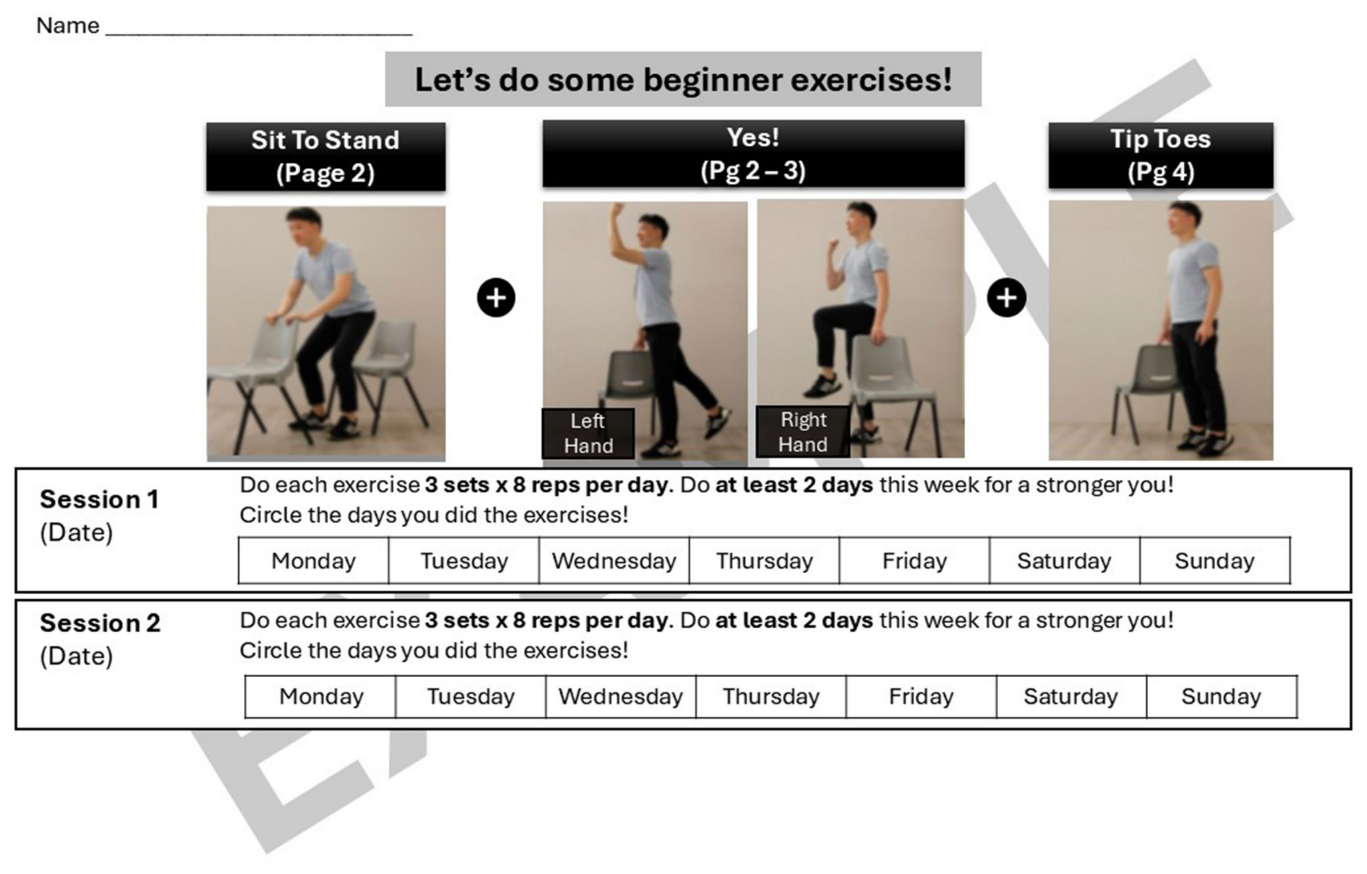
Example of “Steady Lah” homework exercises—tracking sheet.

**Figure 4:**
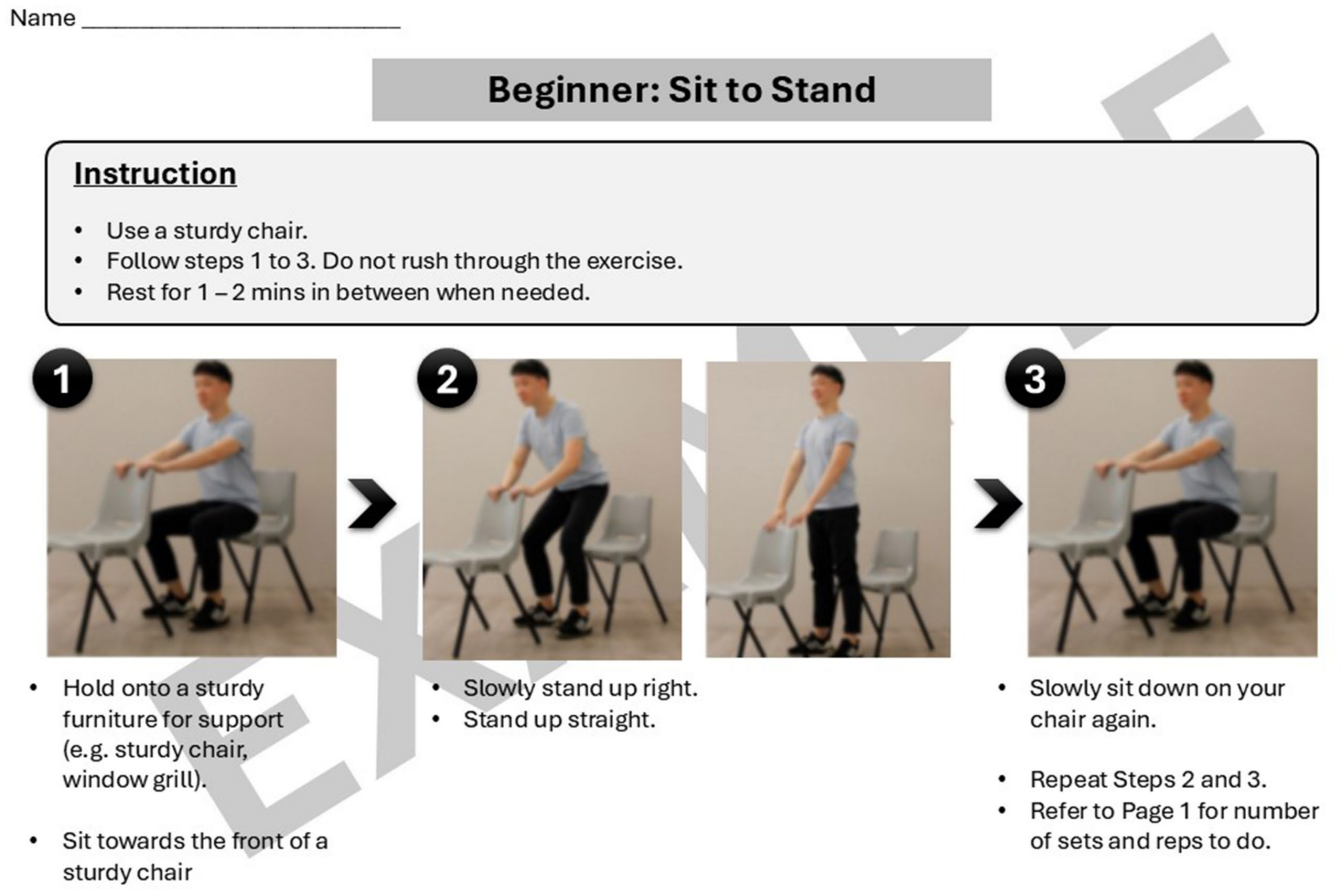
Example of “Steady Lah” homework exercises—English translation.

**Figure 5:**
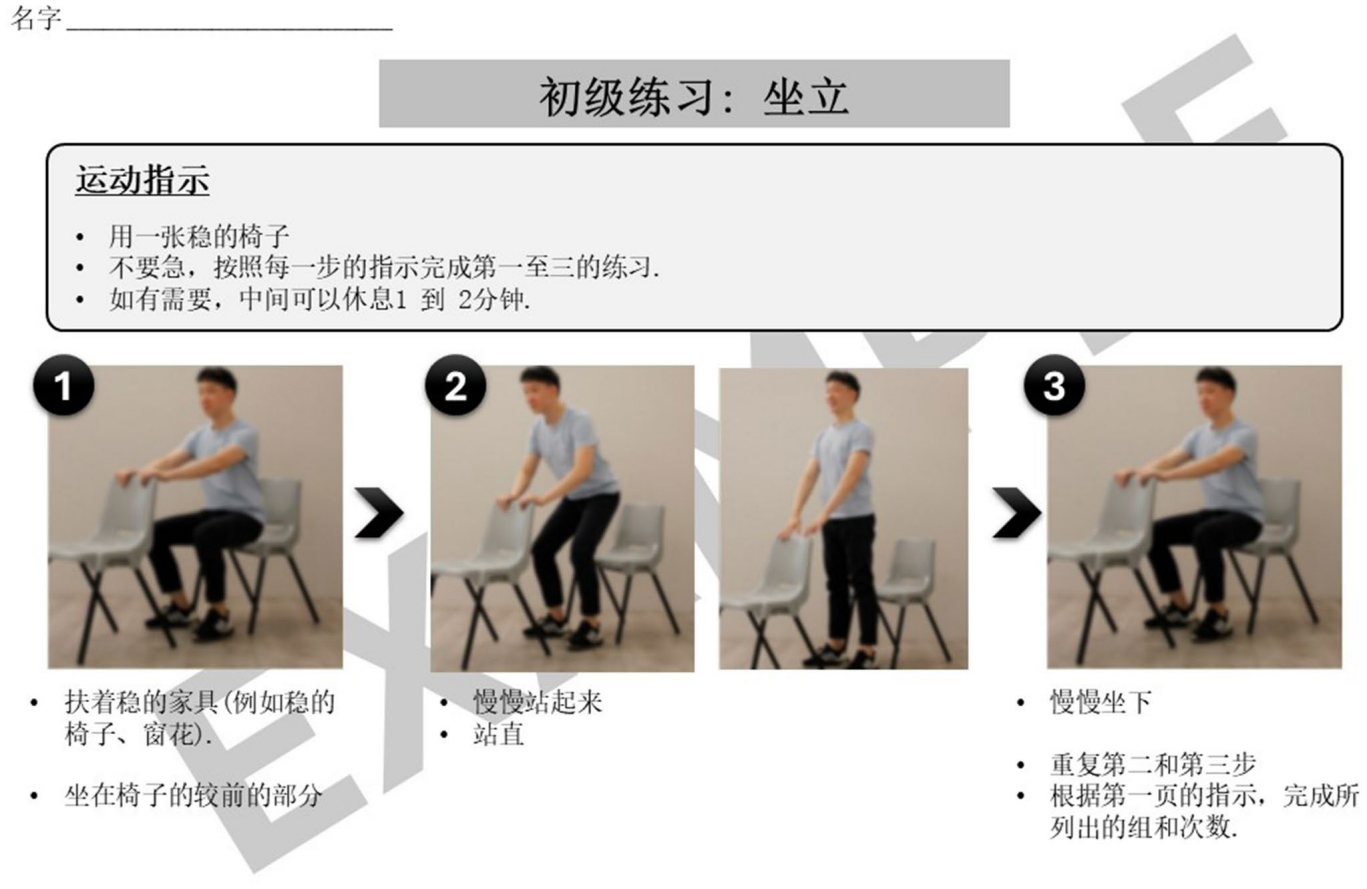
Example of “Steady Lah” homework exercises—Mandarin translation.

**Figure 6:**
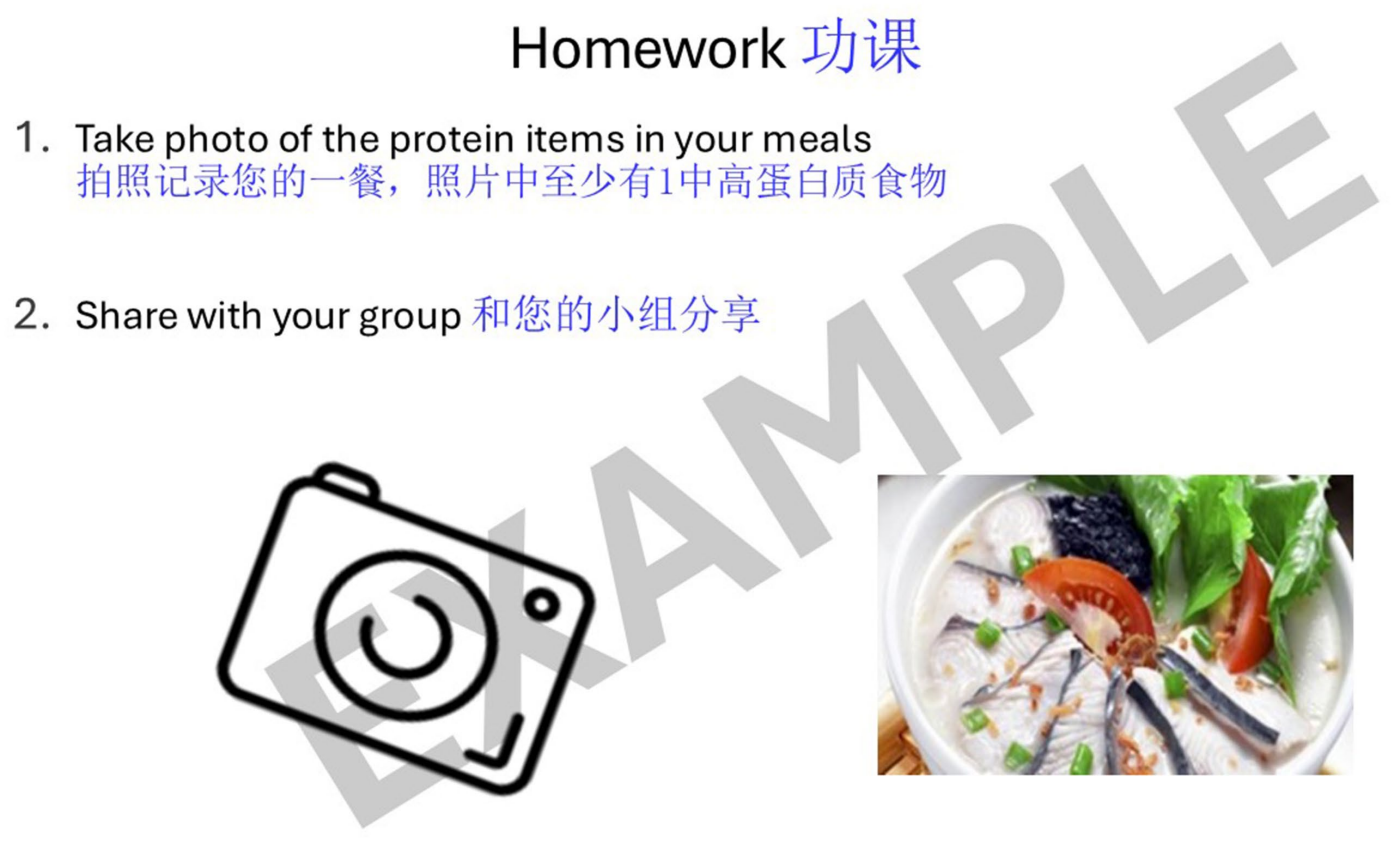
Example of “Steady Lah” homework activity on nutrition.

Lastly, the programme included a homework component where participants were provided with a set of instructions on how to continue with the exercises at home. Figure 3 is an example of a set of homework exercises that participants can perform and track during the days between the “Steady Lah” sessions. For each exercise prescribed, additional material is provided with step-by-step pictorial instructions on how to perform the movements correctly and safely. Figures 4, 5 are illustrative examples of how participants can perform a sit to stand with hand support in English and Mandarin. Certain homework tasks were also assigned to participants as part of the workshop curriculum. This was deliberately designed to encourage participants to apply lessons learnt during the session to their daily lifestyle and be more aware of their dietary choices. Figure 6 is an example of a simple take-home activity where participants identify protein-rich food items in their meals and are subsequently given the opportunity to share this during the session.

As part of the programme, a functional assessment test was administered at the first and last session for participants to better understand their baseline functional ability and appreciate any changes over the course of the programme. The test comprised of three assessments namely, gait speed, number of repetitions of sit to stand in 30 s and time to complete the four-square step test. These assessments are commonly used in both clinical and research settings.

## 4 Discussion

### 4.1 Modifying the programme for delivery by trained laypersons

The concept of general community-based physical activity programmes for well older adults conducted by fitness trainers is not new in Singapore. However, the unmet need that our programme aims to address is that of delivering an intervention specifically for older adults with early frailty. The profile of older adults with early frailty is notably different from the general older population and fitness trainers are less familiar with engaging this population as compared to healthcare professionals. However, in Singapore’s context, a model anchored on delivery by specialised healthcare professional manpower would not be scalable due to both cost and scarcity.

To address this gap between fitness trainers’ competencies and the higher level of needs among older adults with early frailty, we adopted the following modifications, in consultation with the experts from TTSH. The overall principle was to identify and retain the core elements of the programme and remove or simplify other elements that were more challenging to administer. We reduced the complexity of both the physical activity and nutrition-based components to remove any highly technical movements and content that could not be delivered by individuals without formal healthcare training. A defined curriculum with a fixed number of exercises and adjustment of the difficulty tiering of the exercises such that fitness instructors could offer progression or regression based on the participants’ profiles and progress was developed. In addition, a standardised set of educational material for the workshop was also developed with emphasis on pegging the difficulty level of the content to that of a non-healthcare professional.

### 4.2 Assessing effectiveness

The follow-up question that comes with making any modifications to a previously proven intervention is that of sufficient fidelity and the ability for the modified programme to still realise the intended outcomes. Given that the overall objective of this pilot was to test out a modified model of programme delivery and assess if the processes were feasible to scale, the ability of this study design to offer robust effectiveness evaluation data was limited. The overall approach adopted involved comparison of participant assessment scores before and after completion of the programme to assess if the participants recruited in this pilot saw any benefits and improvements at the individual level. A paired *t*-test was used to assess the statistical significance at a *p*-value of 0.05, and all statistical analysis was run on STATA 13.0.

Overall, statistically significant improvements were observed among participants for both functional assessment test scores and protein and calcium intake after completion of the programme. Given the earlier limitations mentioned, these results serve to provide an initial sensing of the potential effectiveness of the programme and will need to be triangulated with more data, when available.

**Figure 7:**
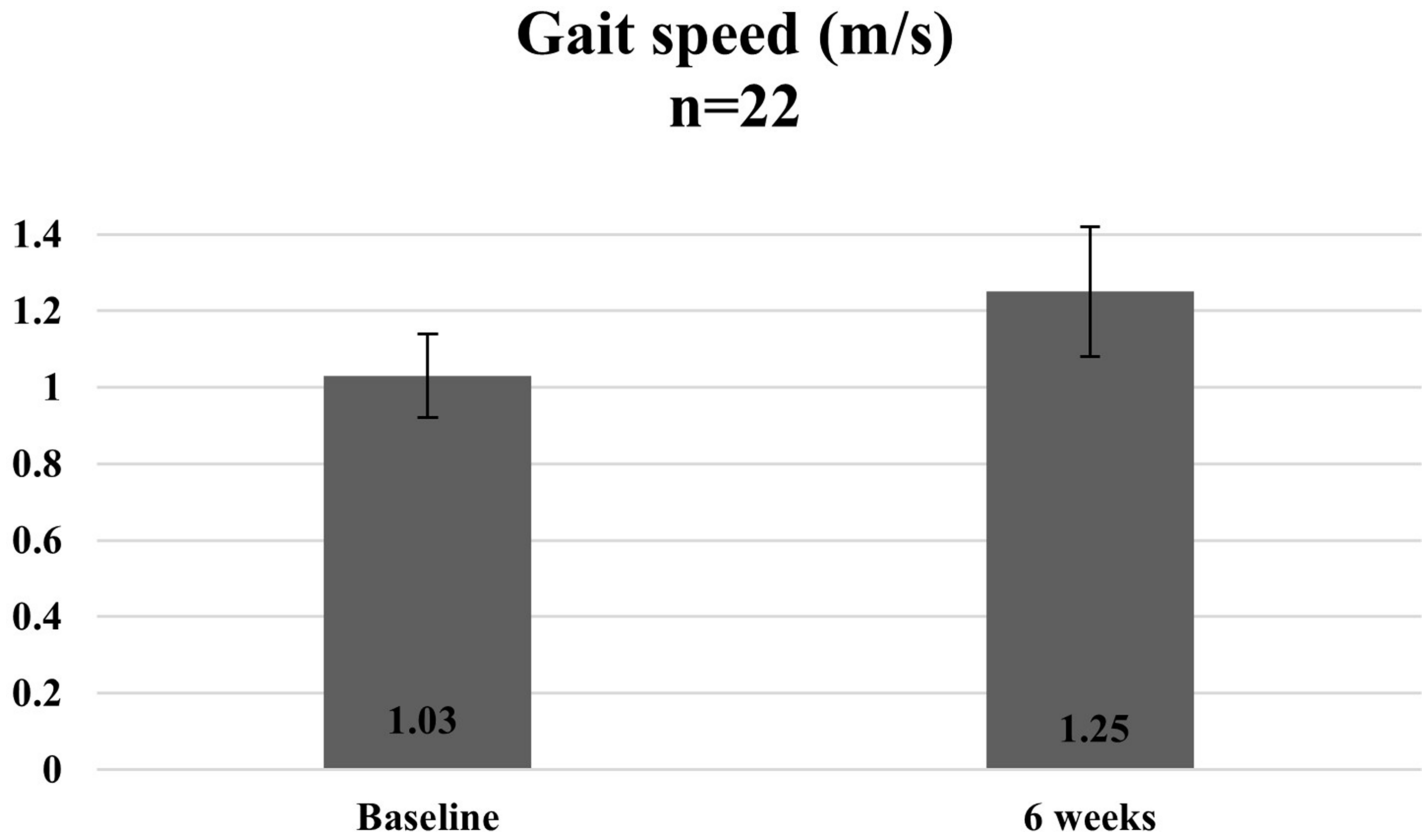
Average gait speed of participants at baseline and 6 weeks.

**Figure 8:**
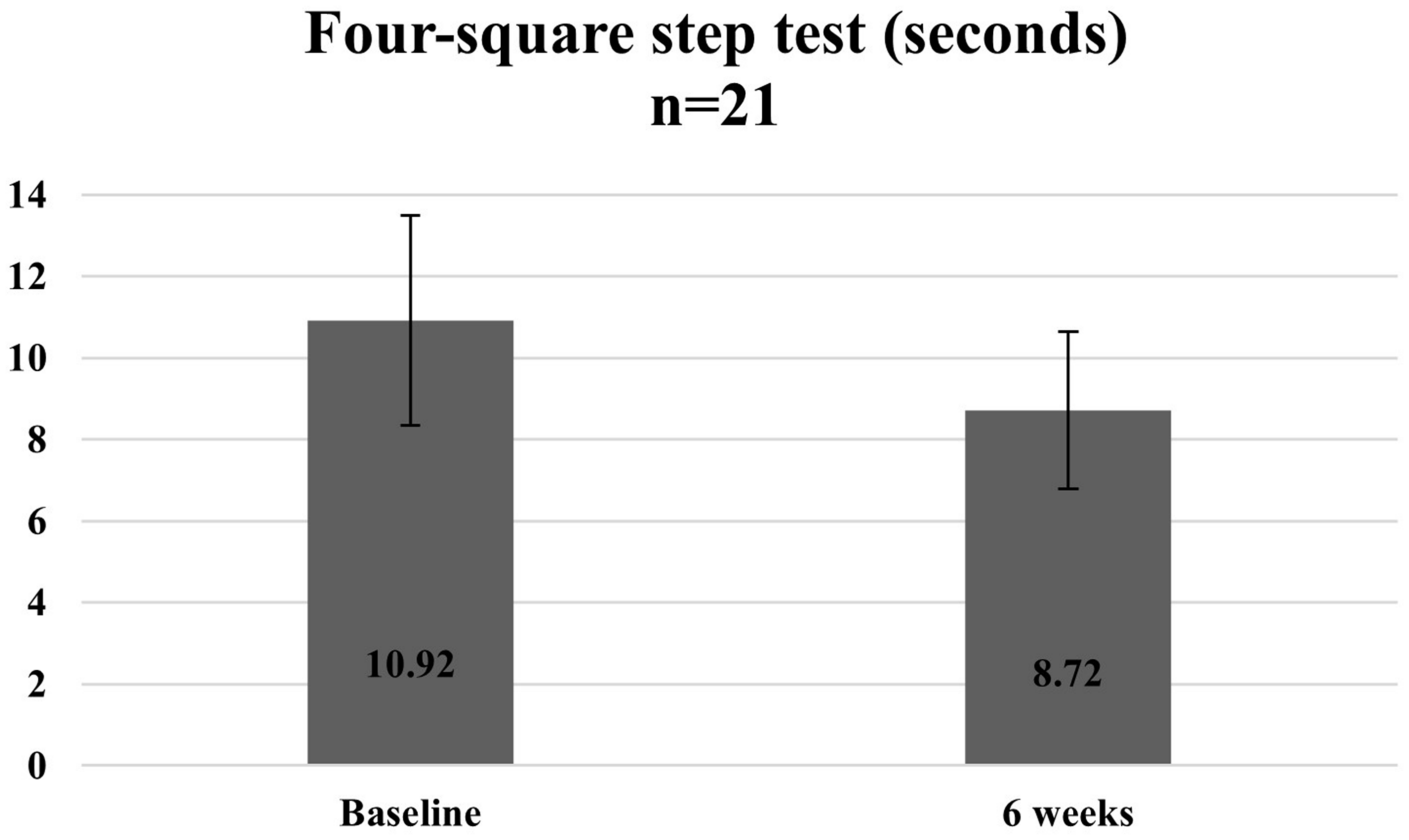
Average time taken to complete the four-square step test at baseline and 6 weeks.

**Figure 9:**
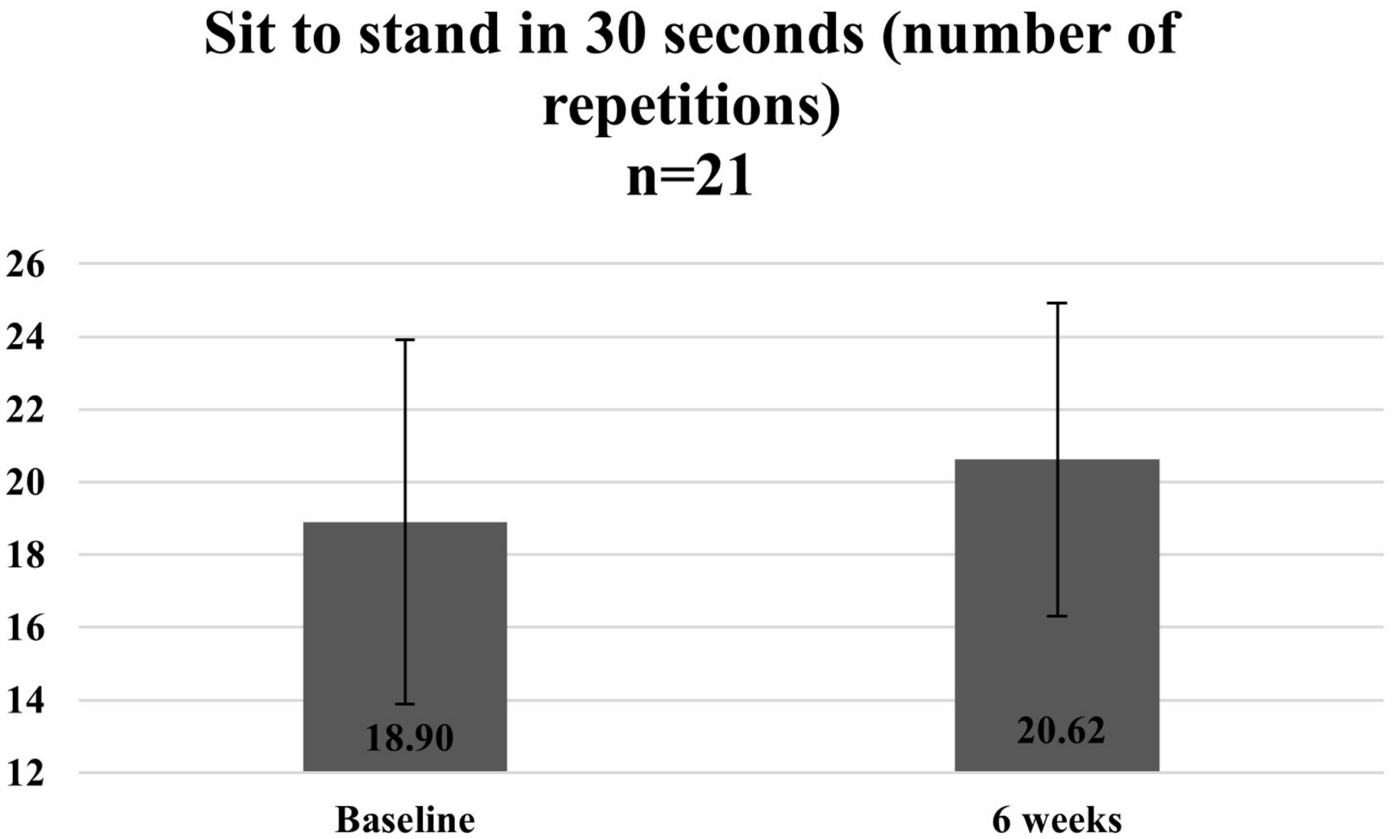
Average number of repetitions of sit to stand in 30 s at baseline and 6 weeks.

Figures 7–9 illustrate the observed change in the average functional assessment scores before and after completion of the programme. Out of the 23 participants enrolled, 22 participants attended the follow-up session where the functional assessment tests were repeated. Out of the 22 participants, one declined to participate in the 30 s sit to stand and the four-square step test.

**Figure 10:**
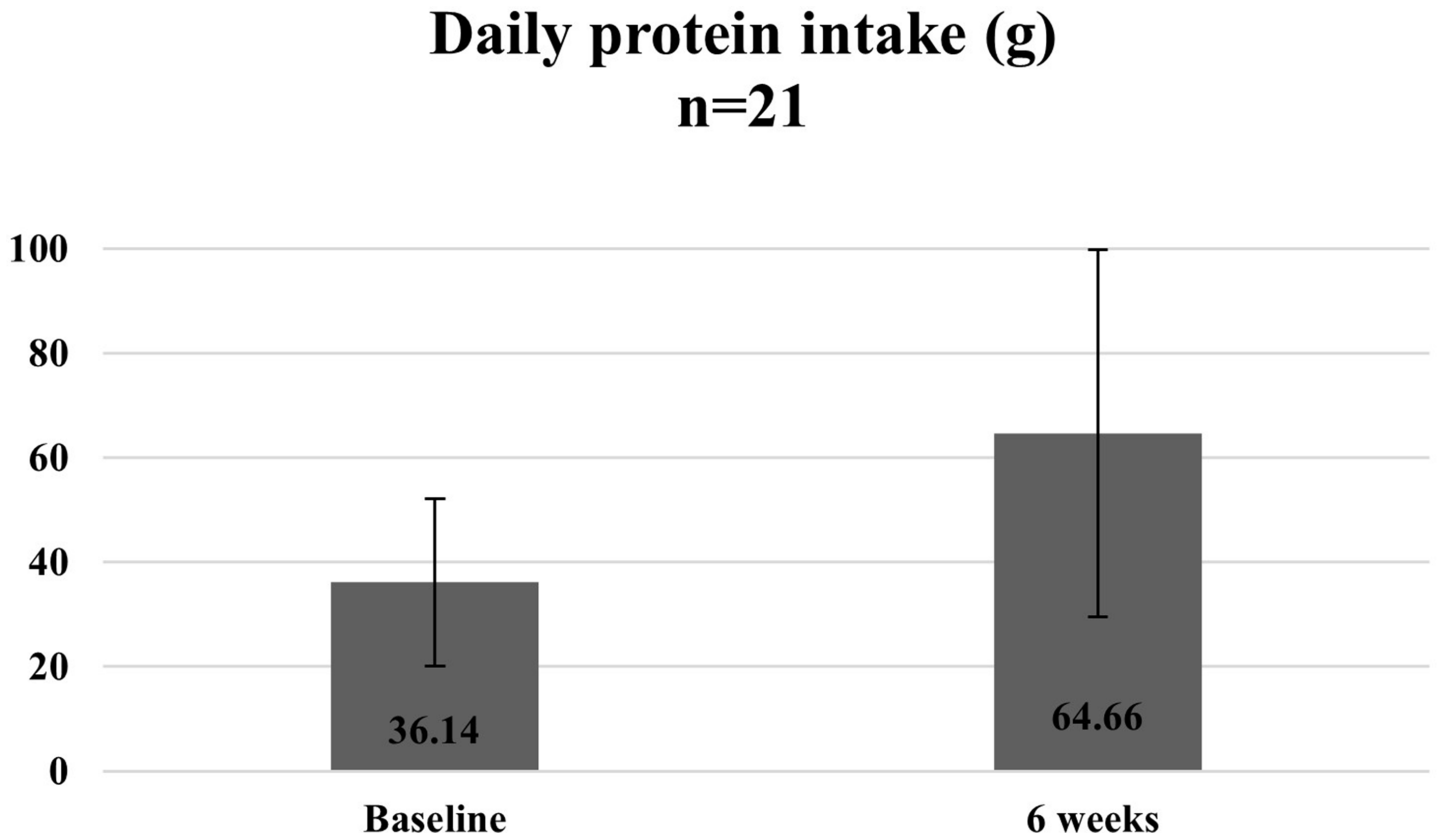
Average daily protein intake at baseline and 6 weeks.

**Figure 11:**
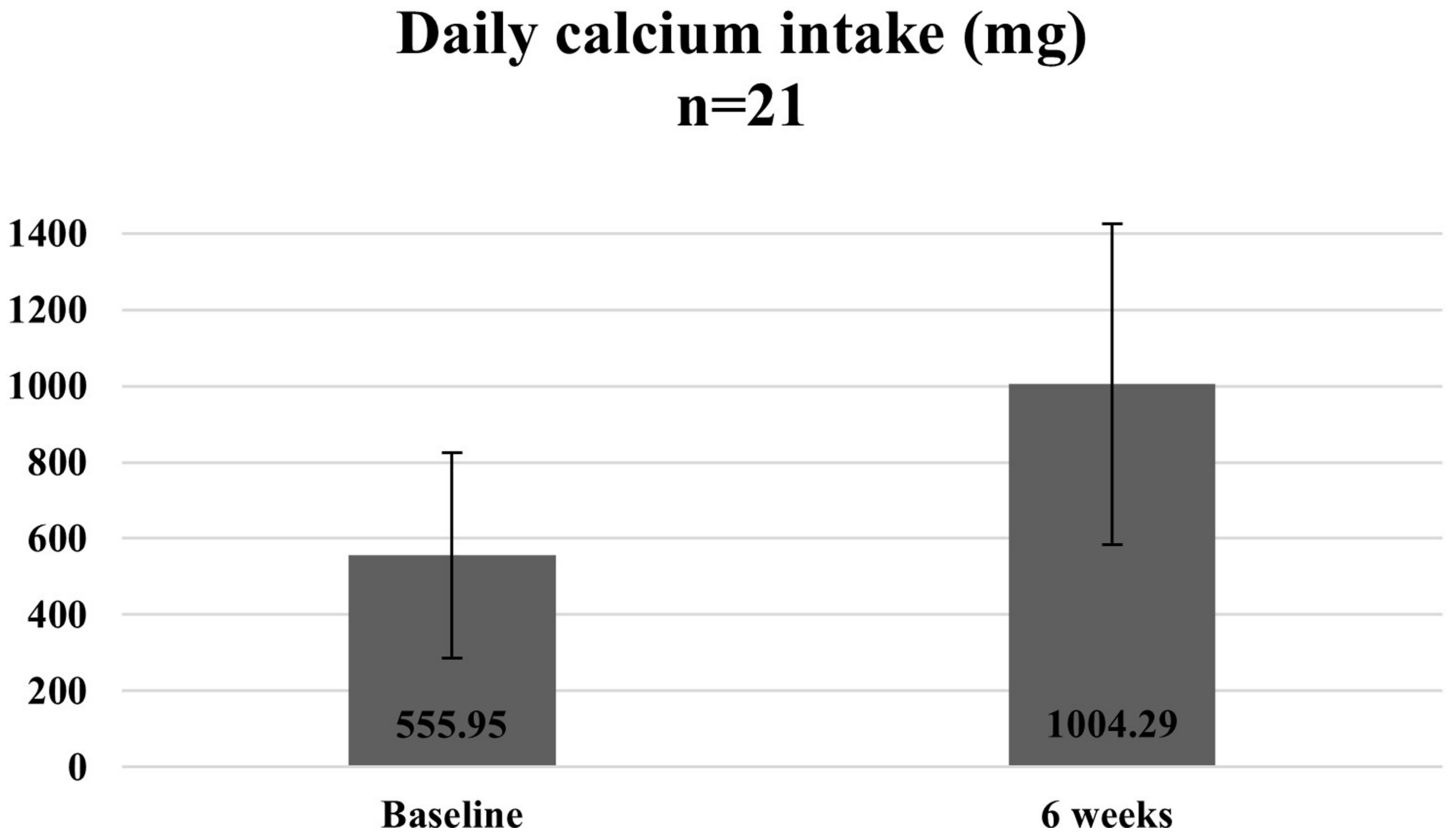
Average daily calcium intake at baseline and 6 weeks.

**Figure 12:**
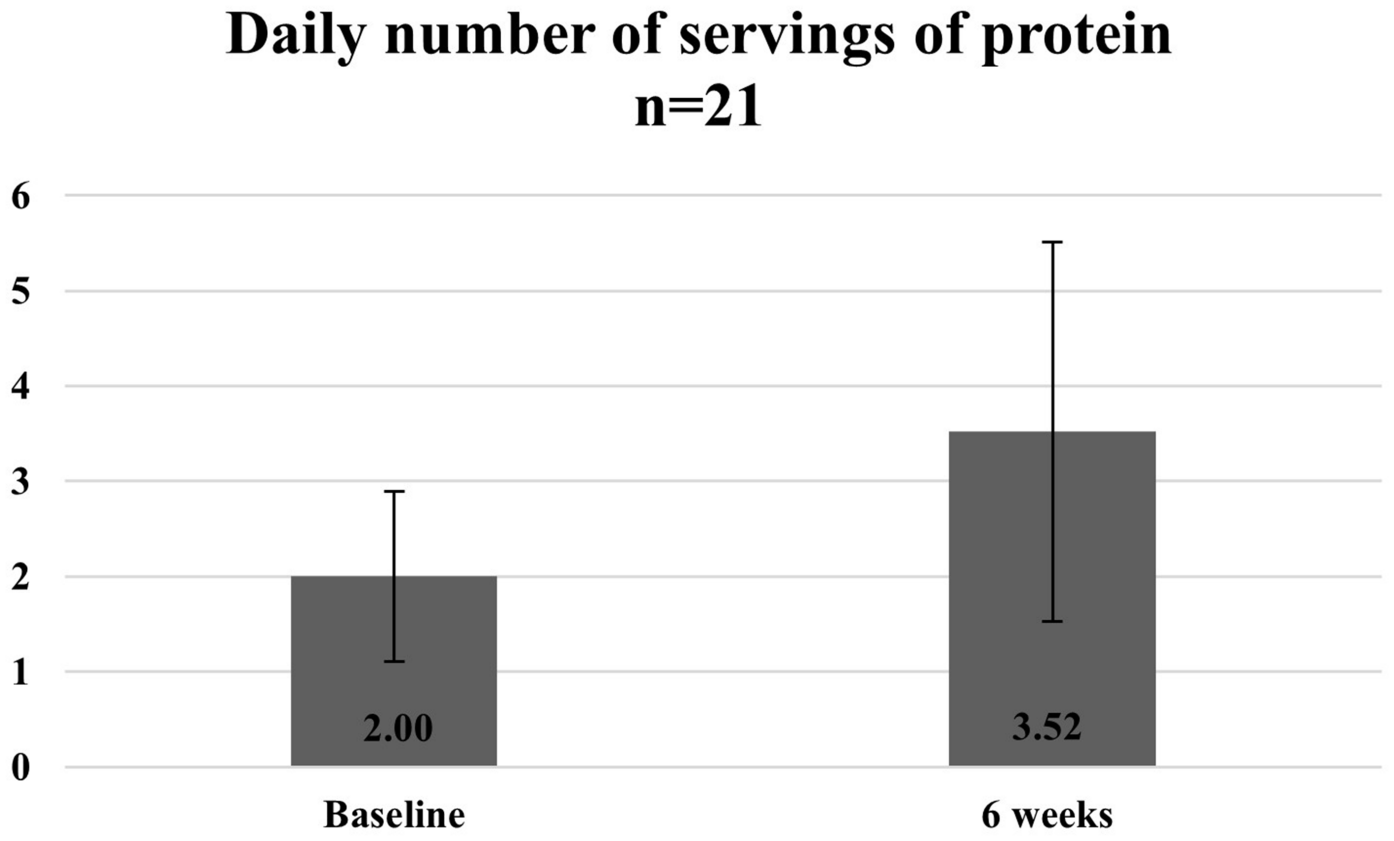
Average number of servings of protein at baseline and 6 weeks.

Figures 10–12 show the changes in average protein and calcium intake at baseline and after completion of the programme. The data was available for 21 participants.

### 4.3 Addressing safety considerations

Another important consideration specific to programmes involving physical activity and special populations like older adults is safety. In the shift from a healthcare professional-led model of delivery to a trained layperson-led model, we noted that additional measures would have to be taken to address participants’ safety considerations. Some of the measures that we adopted include placing significant emphasis on safety during instructor training, restricting the class size to support a high instructor to participant ratio and performing pre-participation suitability checks to ensure that participants can perform the exercises in the programme curriculum. For example, participants who report any signs and symptoms suggestive of cardiovascular disease are advised to proceed for a medical consultation prior to joining the programme. During the set-up and administration of the four-square step test used to assess dynamic balance, additional emphasis is placed on safety as participants are required to change direction and walk backwards. At any point in time, two fitness instructors are required at this test station to ensure that participants perform the test safely. In addition, participants who require the use of walking aids or have unsteady gait are exempted from this test. In the pre-programme training, our fitness instructor was also equipped with the knowledge on how to recognise participants who were unwell or unsteady when performing exercises such that he could intervene early before any potential incidents.

Throughout our planning, conduct and review of the pilot run, the team had frequent discussions related to optimising the safety processes of the trained layperson-led model of “Steady Lah.” While we acknowledge the need to deliver the programme safely, we noted that a balance needed to be struck between introducing multiple new safety processes, minimising complexity of programme administration from the instructor’s perspective and ensuring that the barrier to entry to the programme from the older adults’ perspective was not too high that they would be put off from enrolling in “Steady Lah.” In the absence of clear guidelines on recommended safety processes for community-based early frailty interventions, our experience suggested the need for constant iteration to best address the competing interests of the stakeholders involved in any programme.

### 4.4 Reaching the target population adequately

The overall aim of our programme is to address the unmet needs of community dwelling older adults with early frailty. For this run of “Steady Lah,” we tapped on the Active Ageing Centre and its existing clientele to identify suitable older adults. This was an opportunistic group of participants. However, from our experience we observed that it was challenging to identify more potential participants with early frailty who may benefit from the programme based on this outreach approach. Out of 34 older adults shortlisted by the AAC manager, only about two-thirds met the programme inclusion criteria. Also, the participants were predominantly female. One of our hypotheses are that older adults with early frailty may not have found the previous general physical activity programmes suitable for them and hence may not be regular participants in the AAC activities. Hence, there is a need to diversify the recruitment strategy for “Steady Lah” to truly reach out to all potential participants who could benefit. For example, future iterations may need to work with the Silver Generation Office, a national volunteer outreach group that engages with older adults or engage healthcare institutions to receive referrals from healthcare providers such as general practitioners and community nurses (14).

## 5 Limitations

The focus of the experience documented in this paper was to modify and implement a community-based frailty intervention programme that was previously delivered, solely by healthcare professionals. The emphasis was on understanding if the modified programme could be implemented by trained laypersons and if there were any additional considerations that needed to be addressed to ensure that the programme benefitted older adults with early frailty in the community. The overall intent in the long run is to run “Steady Lah” at multiple sites throughout Singapore. As such, this was not intended to be a robust programme evaluation and lacks the methodological rigour to do so. The absence of a control group makes it harder to fully appreciate and size the impact of the intervention on the outcomes. Also, the convenience sample of participants from the Active Ageing Centre was not representative of the broader population of older adults with early frailty. As our team iterates and implements the programme at more sites, conducts systematic training for cohorts of fitness instructors conducting the programme and reviews the experiences from those runs, additional data and lessons can be assessed to offer more insights. As of August 2023, “Steady Lah” has been progressively implemented at more community sites throughout Singapore with the support of community partners and a growing pool of fitness instructors with special training in managing older adults with early frailty (15). The evaluation of these additional runs is ongoing.

## 6 Conclusion

In conclusion, our experience from this inaugural run of a community-based frailty intervention programme for older adults with early frailty, delivered by trained laypersons, demonstrates that this sub population of older adults can indeed be better supported in the community by non-healthcare professionals. In Singapore, evidence-based programmes targeting this sub population are initially developed in healthcare institutions but subsequently require support and resources from the community to expand the programme reach. This requires good collaboration between stakeholders in healthcare institutions and the community to co-develop a model that ultimately prioritises the needs of frail older adults without compromising on the quality of the intervention. Other communities embarking on a similar journey to support older adults with frailty in the community can consider taking reference from this case study to design and implement community-based interventions.

## Data Availability

The raw data supporting the conclusions of this article will be made available by the authors, without undue reservation.
